# Design Considerations of an Analog Voltage Mode Readout Circuit for the CMOS-SOI-MEMS Gas Sensor Dubbed GMOS

**DOI:** 10.3390/mi16060658

**Published:** 2025-05-30

**Authors:** Efraim-Lavi Bukshish, Sharon Bar-Lev, Tanya Blank, Yael Nemirovsky

**Affiliations:** Electrical and Computer Engineering Department, Technion—Israel Institute of Technology, Haifa 32000, Israel; bukshishe@campus.technion.ac.il (E.-L.B.); sharonb@technion.ac.il (S.B.-L.); tblank@technion.ac.il (T.B.)

**Keywords:** gas sensor, CMOS–SOI–MEMS, ROIC, TMOS, GMOS, MOS transistors, voltage mode, chiplet

## Abstract

Modern gas sensor technology is becoming an important part of our lives. Hence, there has been considerable effort over the past 25 years towards the goal of creating low-cost gas sensors by employing modern microelectronics technology to manufacture both the sensing element and the signal conditioning circuitry on single silicon chips. CMOS sensors based on CMOS-SOI-MEMS technology seemed to be a good candidate for the monolithic approach. In this study, we critically review this approach. We show the advantages of chiplet-based designs for gas sensors that are based on CMOS-SOI-MEMS technology, dubbed GMOSs. The design of a monolithic GMOS system based on the voltage mode reading of a GMOS transistor connected in a three-terminal configuration is presented and validated for the first time. This study led to the understanding that a chiplet-like design should be preferred since the sensor and the readout circuitry of traditional gas sensors exhibit conflicting technological requirements. The innovation of this work is both in the readout design that it posits and in the resulting paradigm shift.

## 1. Introduction

Modern gas sensor technology is becoming an important part of our lives. Hence, there has been considerable effort over the past 25 years towards the goal of creating low-cost gas sensors by employing modern microelectronics technology to manufacture both the sensing element and the signal conditioning circuitry of a single silicon chip [1,2,3,4,5]. CMOS sensors based on CMOS-SOI-MEMS technology seemed to be a good candidate for the monolithic approach [6,7]. In this study, we critically review this approach and show the advantages of chiplet-based designs [8].

Recently, two novel uncooled sensors based on a CMOS-SOI-MEMS process were invented at the Technion. The first is a thermal sensor named TMOS (T for thermal) and it is based on a suspended micro-machined transistor fabricated using CMOS-SOI technology. It undergoes a post-processing release through dry etching. The thermally isolated transistor, which operates at subthreshold, can convert IR radiation power to an electrical signal with high efficiency. The temperature of micromachined MOSFET transistors which absorb IR photons increases and, while operating at subthreshold, exhibits an exponential dependency on the I-V characteristics of the sensor. The TMOS is currently manufactured commercially under a licensing agreement [9].

The second sensor is dubbed GMOS (G for gas) and is based on the formerly described thermal sensor, the TMOS. The GMOS is a gas sensor which uses the TMOS’s principles to detect specific gases in the air. This sensor’s operation relies on the combustion reaction of analyte gases that occurs on a catalytic layer: the exothermic reaction releases heat and increases the transistor’s temperature. A change in temperature will vary the I-V slope on the same basic working principle of the TMOS, indicating the presence of gases. The TMOS’s high temperature sensitivity is the backbone of the GMOS’s sensitivity. However, while the TMOS senses electromagnetic radiation, the GMOS senses the heat of chemical reactions [10,11]. The released heat of the reaction is proportional to the gas molecule concentration. The measurement is performed in a differential mode between the active pixel, which is covered with a catalyst, and the blind pixel without a catalyst. The function of the GMOS readout is to accurately measure the transistor’s response to the gas concentration.

**Figure 1:**
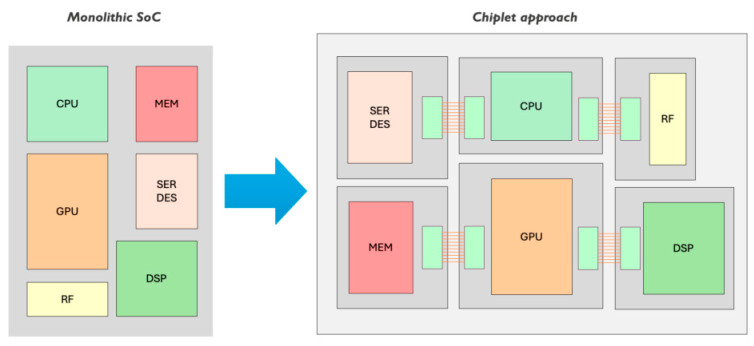
Chiplets offer a modular system that combines separate chips from different vendors and technology nodes, instead of designing all functions into one monolithic system on the chip [8,12].

The key to understanding the retraction from the monolithic approach to the hybrid approach, as shown in Figure 1, is the following: It is well-known that sensors’ operating principles are often based on parasitic effects. Hence, the optimal CMOS technology for GMOSs is the older CMOS technology, while the optimal CMOS readout technology is a more advanced technology.

In Section 2, we present the older CMOS technology that is optimal for use in GMOS sensors. The key parameter for GMOS sensors is the value of the threshold voltage and their dependency on temperature—the larger the better. This is in contradiction to the VLSI design requirements, which prefer a lower Vt to decrease the power and achieve high uniformity in the Vt.

In Section 3, we present the more advanced CMOS technology node, which enables us to improve the readout circuitry based on the voltage readout of a GMOS with three terminals.

In Section 4, we validate the design consideration by showing our preliminary gas detection results.

Section 5 summarizes the work and recommends a hybrid packaging of the analog readout with the sensor, like in a chiplet.

Section 6 concludes the main results.

## 2. The GMOS Sensor and the GMOS Innovation

The beauty and magic of our GMOS sensors are in the technology innovation that they represent [10,11].

We start with a standard microelectronics wafer, offering mass production at low cost. We use the building block of all silicon chips—the transistor—as the sensor. We transform the transistor into a gas sensing device by adding micromachining and printing a catalytic layer on top. The heating resistor was implemented to be able to increase the temperature of the catalyst before ignition (chemical oxidation reaction). Thus, the GMOS sensor pixel is a 213 × 213 μm^2^ membrane from a CMOS plate which contains a sensitive transistor and a heating resistor and is encapsulated with silicon oxide. A catalytic layer is deposited on the top of the membrane.

Catalysts are substances that speed up a reaction but are not consumed by it and do not appear in the net reaction equation. The marriage of chemistry and electronics enables us to ignite the analyzed gas with oxygen in the air. Finally, we detect the heat of the reaction, which is a fundamental physical property.

The outcome is a tiny, low-cost, low-power sensor that attains the following: (i) selectivity/specificity for ethylene, for example, even in the presence of other gases, (ii) high sensitivity, enabling the sensing of a small concentration of ethylene, and (iii) high reliability and robustness in the field and mobile applications.

Combining the chemical and electrical aspects produces the GMOS’s voltage $v_{signal}$ and sensitivity $S_{chemical}$:

(1) $$v_{signal}[V]=-\frac{q}{n\lambda kT}\left(\frac{dV_{t}}{dT}+\frac{{V_{gs}-V}_{t}}{T}\right)\cdot C_{G}\cdot {\left(\frac{1}{K_{S}}+\frac{\delta }{D}\right)}^{-1}\cdot Area\cdot \left(\frac{\Delta H_{C}}{G_{TH}\cdot N_{A}}\right)$$

(2) $$S_{chemical}[\frac{V}{ppm}]=\frac{v_{signal}}{C_{G}}=-\frac{q}{n\lambda kT}\left(\frac{dV_{t}}{dT}+\frac{{V_{gs}-V}_{t}}{T}\right)\cdot {\left(\frac{1}{K_{S}}+\frac{\delta }{D}\right)}^{-1}\cdot Area\cdot \left(\frac{\Delta H_{C}}{G_{TH}\cdot N_{A}}\right)$$

where *q* is the electron charge, *n* is the slope factor, λ is the short channel modulation parameter, *T* is the transistor’s temperature, $V_{t}$ is the threshold voltage, $V_{gs}$ is the transistor’s gate-source voltage, $C_{G}$ is the ambient gas concentration, $K_{S}$ is the gas reaction rate, δ is the stagnant film thickness, D is the gas diffusion constant, $\Delta H_{C}$ is the combustion enthalpy, $G_{TH}$ is the thermal conductance, and $N_{A}$ is the Avogadro number.

From Equations (1) and (2) it is obvious that a large $\frac{dV_{t}}{dT}\;$ increases the sensor’s performance. During operation, the temperature increases and, to remain at subthreshold, a large threshold voltage is required. Moreover, diving deeper and analyzing the parameters that contribute to the threshold voltage temperature sensitivity in Equations (3) and (4) provides a clearer understanding of the benefits of using an older process node to fabricate the sensors [13].

(3) $$V_{t,nmos}=-\frac{E_{gap}}{2q}+\phi_{f}+\frac{\left|Q_{dep}\right|-Q_{it}}{C_{ox}}=-\frac{E_{gap}}{2q}+\phi_{f}+\frac{2\sqrt{\epsilon_{0}\epsilon_{si}qN_{a}\phi_{f}}}{C_{ox}}-\frac{Q_{it}}{C_{ox}}$$

(4) $$\frac{dV_{t}}{dT}\approx \frac{1}{T}\left[\phi_{f}-\frac{E_{g}}{2q}+\frac{1}{2q}\frac{dE_{g}}{dT}\right]\left(2+\frac{\sqrt{\epsilon_{0}\epsilon_{si}qN_{a}\phi_{f}}}{C_{ox}\phi_{f}}\right)\;;\phi_{f}>0$$

where $E_{gap}$ is the silicon’s band gap energy, $\phi_{f}$ is the transistor’s fermi potential, $Q_{dep}$ is the depletion charge, $Q_{it}$ is the interface trap charge, $C_{ox}$ is the oxide capacitance, $\epsilon_{0}$ is the permittivity of the vacuum, $\epsilon_{si}$ is the permittivity of the silicon-oxide, and $N_{a}$ is the acceptors’ concentration.

**Figure 2:**
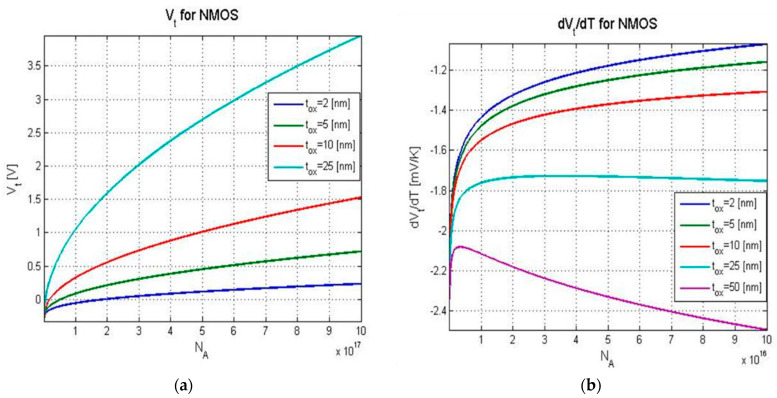
$V_{t}$ (**a**) and $\frac{dV_{t}}{dT}$ (**b**) modeled as a function of the bulk acceptor concentration for an NMOS transistor at 300 K and several gate oxide thicknesses.

$\frac{dV_{t}}{dT}$ and $V_{t}$ are tied to the oxide capacitance, $C_{ox}$. At 300 K, $\frac{dE_{g}}{dT}=-2.7\cdot 10^{-4}[\frac{eV}{K}]$, and, by using Equations (3) and (4), the threshold voltage and its temperature sensitivity were calculated as a function of the bulk acceptor concentration for several gate oxide thicknesses, as presented in Figure 2.

Figure 2 shows that, at a constant number of acceptors $N_{a}$, a higher threshold voltage is achieved with a thicker oxide layer $t_{ox}$

As a result of our findings, the CMOS-SOI process chosen for the GMOS sensors is a partially depleted high-voltage 1 µm technology from XFAB Fab in Germany [14]. It is ideally suited for GMOSs that operate at high temperatures up to 400 °C. The thicknesses of the different process layers are 250 nm and 1000 nm for the device layer and BOX, respectively, and the sensors have low voltage of 5 V at their drain.

To conclude, GMOS sensors have been proposed for applications in agriculture, industry, ecology, and well-being. GMOS sensors were developed to solve the main gas sensing issues such as high sensitivity, cross selectivity, gas mixture analysis, and humidity effects [15,16,17].

## 3. The New GMOS Readout Circuit Based on Voltage Mode, 3 Terminal-Transistor, and the CMOS Readout Technology Under Study

The CMOS readout technology is based on the use of 0.13 µm node and its implementation was fabricated in a different XFAB in France [14].

A robust readout circuit is required for the sensor to operate smoothly. The readout circuit consists of the following:

1. A pre-amplifier in the form of a differential amplifier, with the input devices being two GMOS sensors. Whilst one is an “active” GMOS which contains a catalyst layer to react with the volatile gas compounds, the second is a “blind” GMOS without a catalyst layer that acts as a reference and which cannot change its temperature with the presence of gas;
2. A correction DAC that equalizes the differential amplifier branches’ components (GMOSs and loads) to avoid device variations caused by fabrication, as well as device variations from the sensors’ post-processing;
3. An instrumentation amplifier (differential input to single-ended output) with four different options for gain to further amplify the signal;
4. CMFB (common mode feed-back) to anchor the differential amplifier’s common mode voltage at the output terminal;
5. A precise current reference and biasing circuit that is independent of temperature.

**Figure 3:**
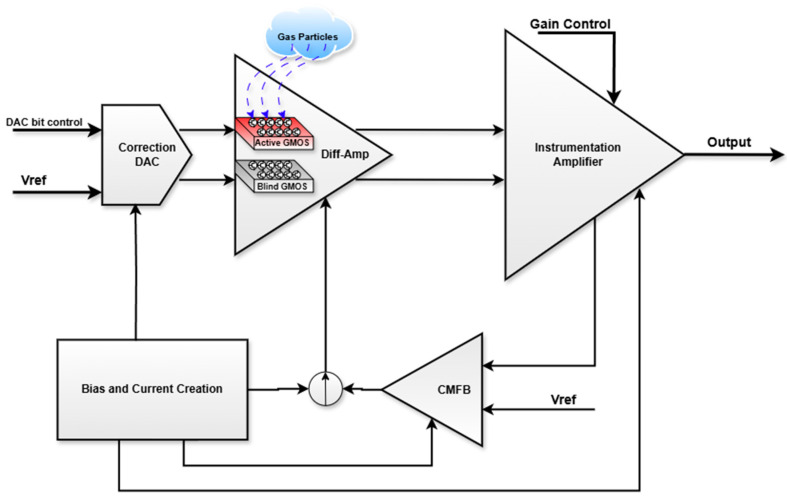
Block diagram of the readout.

The readout integrates all of the above circuits into one system, as seen in Figure 3.

NB—this section contains figures generated from simulations performed using Cadence—Virtuoso studio for analog circuit design [18].

### 3.1. GMOS Sensors as a Differential Pair

The first step in the system’s implementation was to understand the temperature sensitivity of the sensor while connecting it as a differential amplifier constellation. The amplifier’s input devices are an active GMOS sensor with a catalyst layer, which is sensitive to temperature changes due to chemical reactions with the gas, and a blind GMOS sensor without a catalyst layer, which cannot sense the temperature changes due to chemical reactions. The blind GMOS sensor acts as a reference to the ambient temperature. The GMOS sensors create a voltage difference in the branches due to a small difference in their temperatures while nullifying the temperature’s “common mode”. This ensures that the biased temperature from the heating resistors that are heating the sensors to the right operating temperature will not change the output voltage.

**Figure 4:**
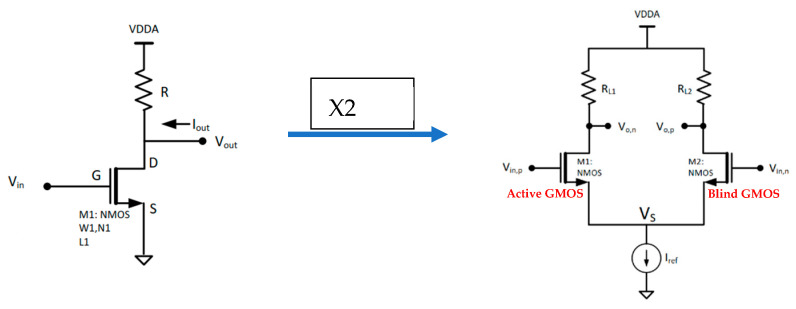
Two common source constellations used to create a differential amplifier.

A simple diff-amp is used, consisting of two transistors in a common source construction using 3T connection (source–drain–gate). The sources are connected to a simple current source made out of an NMOS current mirror to create virtual ground. Each drain is connected to a load, in this case a passive resistor, as shown in Figure 4.

Instead of having a varying input voltage at the gate of the GMOS sensor as the signal to amplify, the gates of the transistors remained constant, while the aim was to sense the temperature change from the chemical reactions of specific gas particles. The change in the output voltage comes from the small changes in the active GMOS’s temperature, as mentioned in Equations (1) and (2).

In addition, since the sensors operate at the subthreshold region, the current source and the resistive loads are adjusted accordingly. The current induced in each branch of the diff-amp is 5 μA; hence, the resistive load is 250 kΩ and the common mode output voltage is 1.25 V (VDD/2). The use of passive resistors provides better linearity as well as independence from temperature variations, in contrast to the use of an active load (PMOS transistors), and also avoids the usual risk of taking the input devices out of the saturation region, since the GMOSs are in subthreshold. The differential amplifier generates a DC gain of 27 dB.

The GMOSs’ input DC voltage is biased to 350 mV at XFAB013 (for a monolithic approach) and to 1.1 V at XFAB018 (for a chiplet approach). This DC input voltage, while inducing 5 μA on each branch, will keep the GMOS transistors at subthreshold. Furthermore, there is a need for a CMFB to anchor the common-mode voltage, since subthreshold systems can be metastable and small changes in voltages and temperatures can create large changes in the output voltage. The CMFB will be reviewed in detail in Section 3.4.

**Figure 5:**
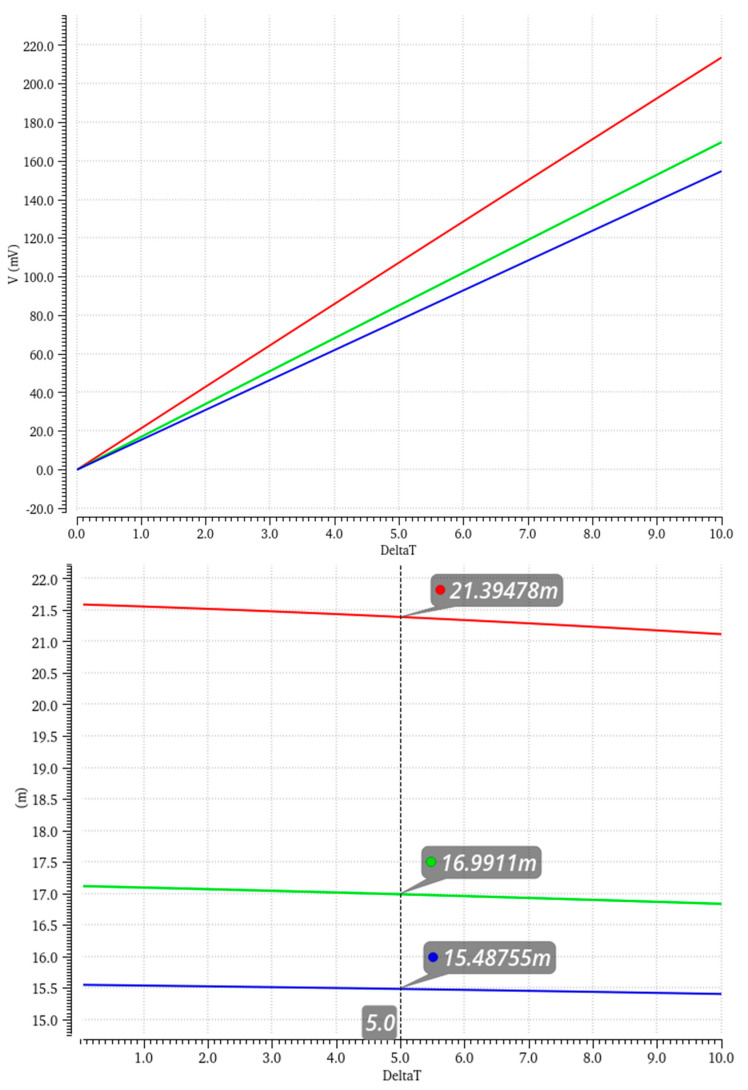
Change in differential output voltage due to temperature change (27 °C (red), 160 °C (green), and 250 °C (blue)) (**top**), derived sensitivity from different GMOS arrays (27 °C (red), 160 °C (green), and 250 °C (blue)) (**bottom**).

Whilst a few different arrays were considered and checked, in order to efficiently use the GMOS stage as well as to uphold the process node limits, it was decided to proceed with 18 parallel pixels of width *W* = 4 × 9.9 μm and length *L* = 5 × 9.5 μm, which, in a typical corner, give an average sensitivity of 21.4 mV/K, 17 mV/K, and 15.49 mV/K for a base sensor temperature of 27 °C (300 K), 160 °C (430 K), and 250 °C (520 K), respectively. Figure 5 presents the output voltage and temperature sensitivity in the temperatures mentioned above and in different process corners.

### 3.2. Correction DAC

**Figure 6:**
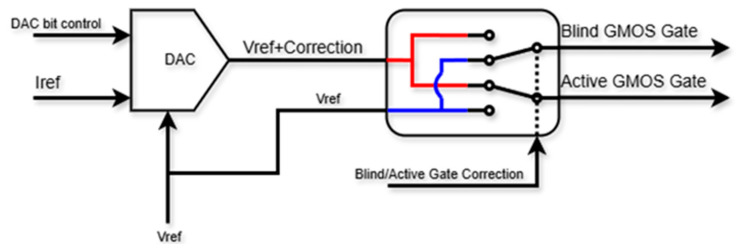
DAC block diagram.

There are multiple uses for DACs in the VLSI world. In the readout circuit used herein, a DAC was used as part of the correction for the sensor’s diff-amp. Since process variations in the fabrication of the resistors and GMOS sensors in the diff-amp circuit are likely, there was a need to negate those effects as much as possible to have two equal diff-amp branches (and hence two equal outputs). The main circuit consisted of a hybrid current mirror, as presented in Figure 6.

Two switches were used, one to choose which sensor (active or blind) would receive the correction voltage, and the other to receive the original reference voltage and peripherals (binary to thermometric decoder and an enabler).

The current mirror was constructed with 4 binary bits and 15 thermometric bits, all of which push current through a load resistor that was connected to the original (external) reference voltage *Vref*. One sensor was connected directly to the *Vref* while the other received “*Vref* + the small correction voltage”. The weaker branch in the diff-amp was the one that received the correction voltage (smaller sensor and/or smaller resistor in fabrication).

**Figure 7:**
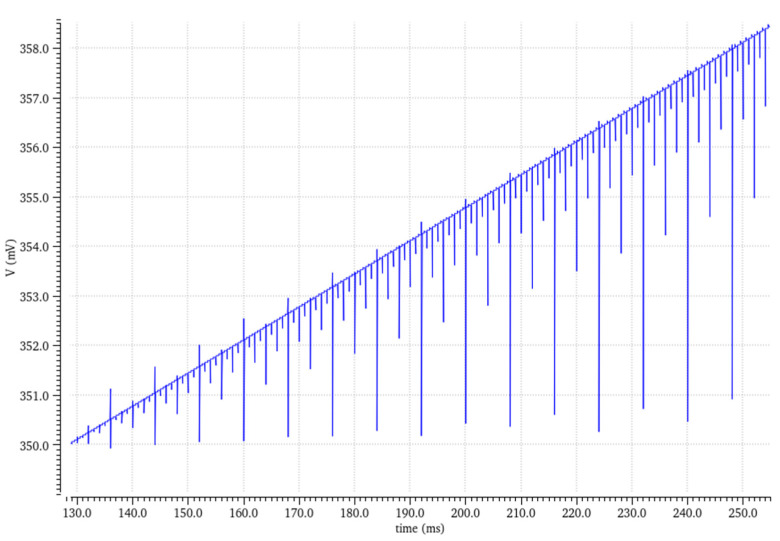
Simulation runs through all available codes.

The output voltage results showed a monotonic increase and a steady incline for both the active and blind outputs, as presented in Figure 7. It was challenging to measure each individual bit manually; however, checking the DNL (differential non-linearity) and INL (integral non-linearity) indicated if there were missing bits or if the graph was not monotonic.

The DNL calculated from the simulations, in the worst-case scenario, was 30% LSB, whilst the vast majority was ~10% LSB. The INL check showed a similar pattern, ensuring that the DAC’s output was monotonous.

### 3.3. Instrumentation Amplifier

**Figure 8:**
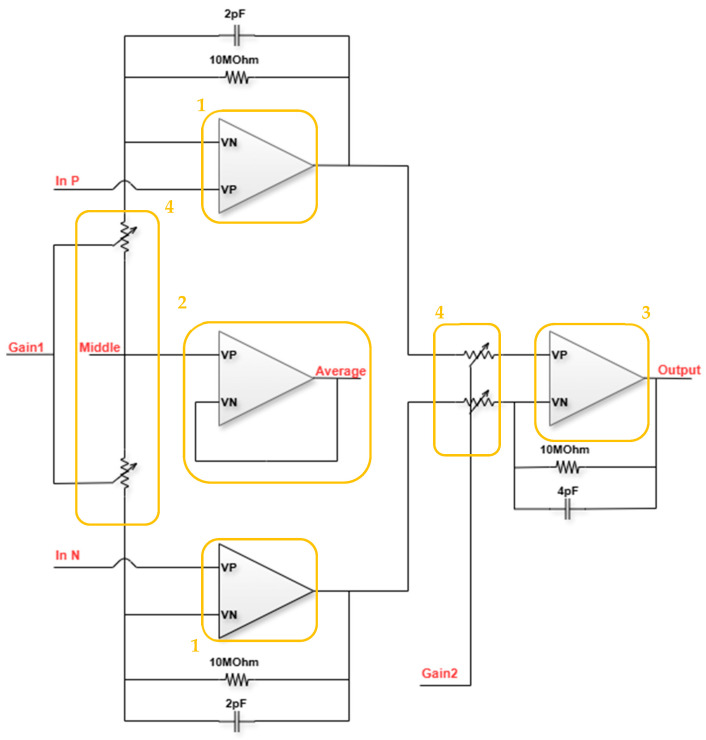
Instrumentation amplifier block diagram.

The design of the circuit’s inst-amp, shown in Figure 8, uses three smaller op-amps in a closed loop constellation for signal amplification and a fourth one for monitoring the average voltage and common mode feedback (CMFB). The op-amps can be divided into the first-stage op-amps (1), the op-amp that is used as a buffer for monitoring and sampling the CMFB signal (2), the second-stage subtractor op-amp (3), and the first- and second-stage gain controls (4).

**Figure 9:**
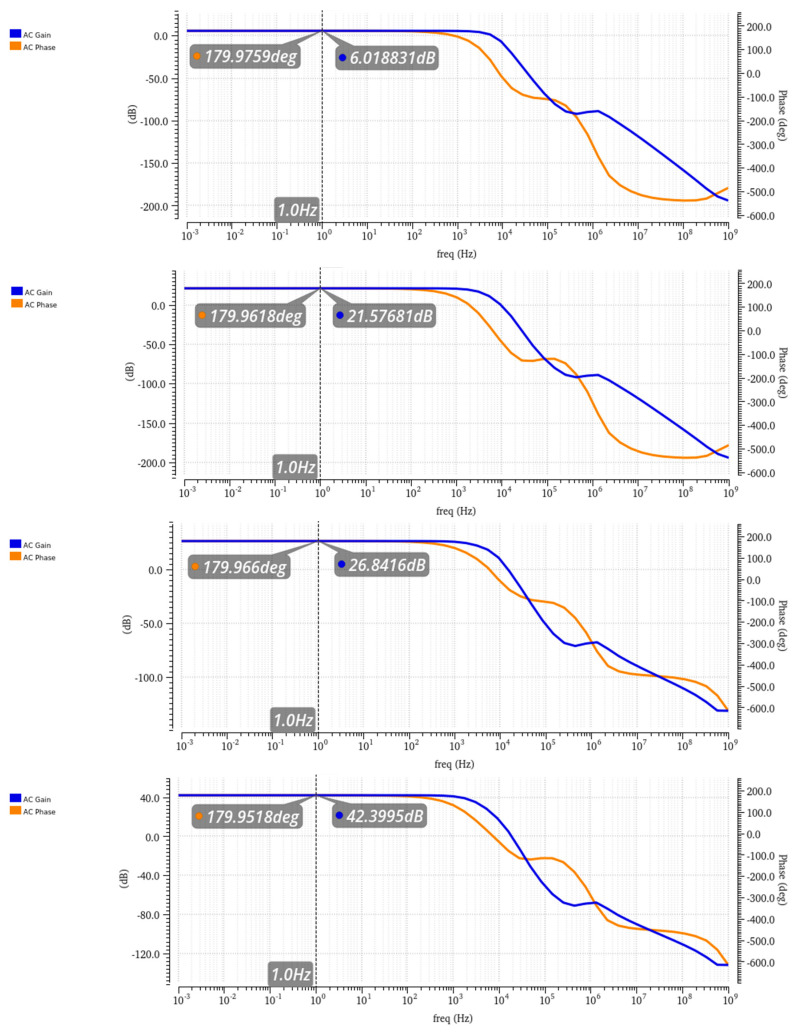
AC gain and phase of the different gain options (marked at 1 Hz).

The first and second-stage op-amps are configured in a closed loop, with the resistors serving as separate gain controls, providing the opportunity to select one 10 MΩ resistor or to connect another 1 MΩ in parallel, with a total resistance of 909 KΩ. The options will be a gain of 2 or 12 for the first stage and 1 or 11 for the second stage (subtractor stage). Based on this design, there are four options to choose from for the total inst-amp gain: 2, 12, 22, and 132 (6 dB, 21.5 dB, 26.8 dB, and 42.4 dB) (shown in Figure 9). The different gain options provide the flexibility to sense a wide variety of concentrations without the risk of reaching the cutoff voltage of the system and losing the signal’s data. For example, lower concentrations will use the high amplification mode of 22-132, while higher concentrations will use the low amplification mode 2-11.

### 3.4. CMFB—Common Mode Feedback

**Figure 10:**
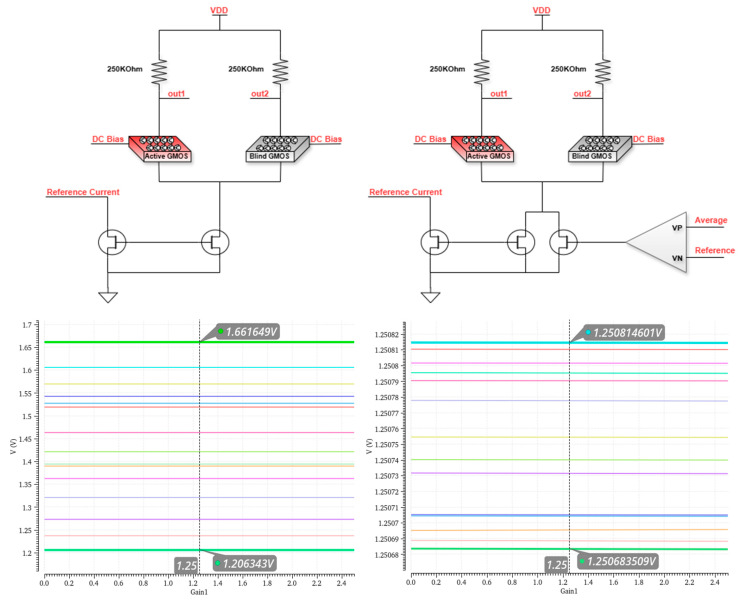
DC common mode simulations without (**left**) and with CMFB (**right**), simulated at different process corners.

While operating the GMOSs in the subthreshold region, it is extremely important to maintain the output voltage common mode. To achieve this, there is a need for feedback: the solution is to integrate the CMFB through the current mirror that connects to the source terminal of the sensors, so that the CMFB will increase or decrease the current that is pulled through the current mirror, creating the required output common mode. The positive input of the CMFB is connected to a reference point, which is the inst-amp monitored net produced at the fourth op-amp. The negative input is connected to an external reference voltage from outside the system. As mentioned above, the output of the amplifier is connected to the current source. Two parallel current sources were used, one constant and the other smaller, and both were controlled by the CMFB to reduce its capacitive load and thereby improve the system’s stability. Figure 10 presents the output common mode voltage of the sensor’s diff-amp without (left) and with (right) a CMFB element simulating different process corners.

The common mode voltage is at an offset of 800 µV at most and a span of 130 µV when the CMFB is incorporated in the circuit, compared to an offset of ~430 mV and a span of 460 mV without the CMFB. With the help of the CMFB, the inaccuracies are kept minimal, considering that the Vref is externally controlled and adjustable.

### 3.5. Bias

**Figure 11:**
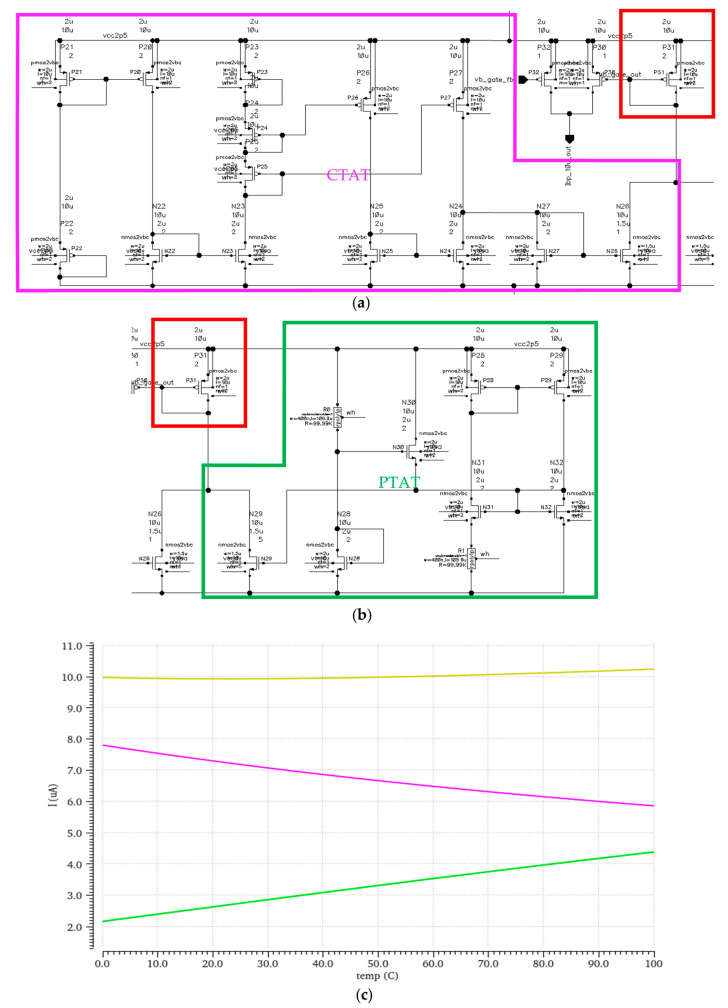
(**a**) CTAT current branch; (**b**) PTAT current branch; (**c**) CTAT and PTAT current output (pink and green respectfully) and combined current output (yellow) in response to temperature.

To produce a precise reference current internally, and with the knowledge that there can be temperature and process corner differences, there is a requirement to use two important elements: a PTAT (proportional to absolute temperature) and a CTAT (complementary to absolute temperature) self-calibrated current reference [19] with a feedback loop. This circuit consists of two parts, one that is in charge of increasing the current proportional to the rise in temperature (PTAT—green frame), and one that is in charge of decreasing the current proportional to the rise in temperature (CTAT—pink frame). Fine-tuning this combination according to the process node used ensures a robust current reference with almost no temperature-dependent current fluctuations, as presented in Figure 11. The PMOS transistor marked in a red square marks the point of reference for the whole circuit.

**Figure 12:**
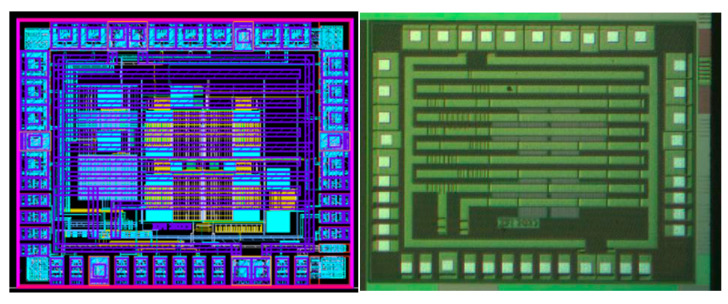
ROIC—layout implementation (**left**) and microscope photo (**right**).

The integration of the circuits mentioned above produced the system’s ROIC. Figure 12 presents the layout implementation and the silicon microscope photo of the ROIC.

## 4. Si Validation

**Figure 13:**
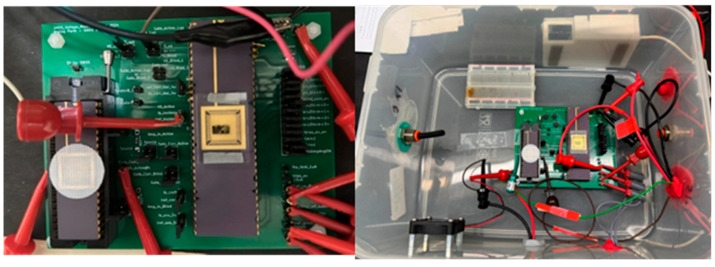
System’s setup inside the sealed container.

In the lab measurements, a precise amount of ethanol was injected into a 6-liter sealed box, creating an ethanol concentration of 14 ppm. Two iterations were made to test all the gains of the system, with the first applying 2.5 V to the heater resistors and the second applying 3 V, thus raising the temperatures to 190 °C and 240 °C, respectively. From one to five test runs were made for every different gain, with each obtaining fairly similar results. Figure 13 presents the setup of the system inside the box, where an external pre-tested GMOS fabricated in an older process can be seen on the left side of the PCB, while the right side of the PCB contains the chip carrier with the new system ROIC (presented in Figure 12). The right picture shows the container with an input tube to pump gas and a fan to circulate the air particles inside it. In addition, an opaque metal plate was added on the box’s seal to prevent light photons from creating additional noise.

**Figure 14:**
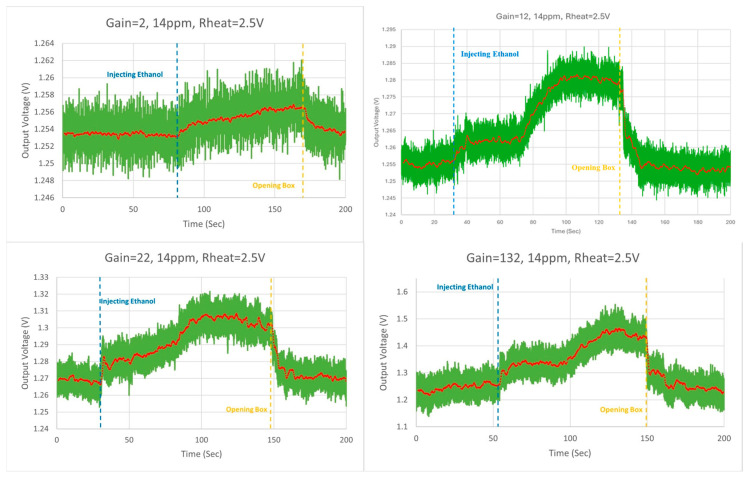
14 ppm ethanol concentration at 190 °C, gain = 2, 12, 22, and 132 measurements.

Figure 14 presents the four different gain measurements (2, 12, 22, and 132) taken while the voltage applied to the heater resistor was 2.5 V. The green curve represents the original measurement, while the red curve represents the measurement’s moving average. Two lines have been drawn on the graphs, representing the moment the ethanol was injected (blue) and the moment the box was opened (yellow).

Extracting the calculations from the measurements at a temperature of 190 °C (heater resistor voltage of 2.5 V) for each gain (2, 12, 22, and 132) produced sensitivities of 243 μV/ppm, 1.52 mV/ppm, 2.73 mV/ppm, and 16.8 mV/ppm and SNRs of 11 dB, 14.43 dB, 17.69 dB, and 16.61 dB, respectively.

**Figure 15:**
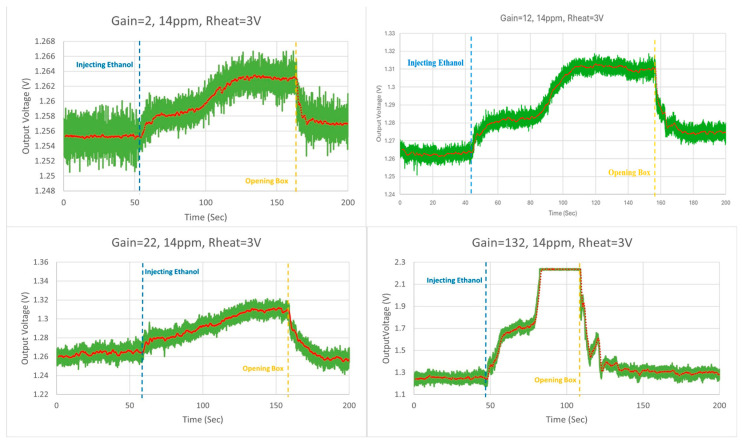
14 ppm ethanol concentration at 240 °C, gain = 2, 12, 22, and 132* measurements.

In the second iteration, 3 V was applied to the heater resistor, which raised the GMOS temperature to 240 °C. Figure 15 presents the measurements taken for the four different gains. Like the previous graphs, the green curve represents the original measurement while the red curve represents the measurement’s moving average. Two lines have been drawn on the graphs, representing the moment the ethanol was injected (blue) and the moment the box was opened (yellow).

The calculations from the measurement of each gain (2, 12, 22, and 132) produced sensitivities of 619 μV/ppm, 3.69 mV/ppm, 5.95 mV/ppm, and 70.46 mV/ppm and SNRs of 20.6 dB, 23.47 dB, 25.19 dB, and 31.04 dB, respectively. At a heater resistor voltage of 3 V, the 132-gain results are marked since the output signal was clipped; therefore, it can be presumed that the sensitivity and SNR would have been higher.

The linear behavior of the sensitivity with different gains allows gas measurements to be taken at various concentration ranges with good accuracy. Moreover, for different applications, different gases, and different concentration ranges, the necessary gains can be selected and the optimal ROIC can be manufactured.

Table 1 and Table 2 summarize the calculated (lab measured) sensitivity and the sensitivity normalized to the actual (lab measured) gain of the two tests. Since the signal was clipped, the 132-gain measurement was not added to Table 2.

In summary, applying a lower voltage of 2.5 V to the heater resistors resulted in lower sensitivity due to the decrease in reaction intensity e. The results confirmed the linearity of the readout circuit, with a 2.5% variation in the sensitivity being normalized to the actual gain. When 3 V was applied to the heater resistors, the sensitivity increased by 250% compared to the previous test. However, achieving consistent linear sensitivity was challenging at the two highest gain settings. At a gain of 22, the sensitivity was lower than expected, while at a gain of 132, the measurement reached the readout circuit’s clipping voltage. Despite these challenges, the readout circuit demonstrated promising performance, providing solid evidence of its robustness.

Table 3 presents a comparison between different gas sensors and the work done in this study, showing promising results.

NB: The noise’s RMS value contains a combination of noises from the GMOS and the readout circuit as well as from the power supplies, long wires, bad connections etc. For this reason, the expectation is a higher SNR than was measured.

## 5. Summary

The more advanced VLSI analog and mixed-design technology of our sensor enables a higher performance readout due to using the 130 nm CMOS process from XFAB [14]. The main features of this technology are a lower oxide thickness and higher doping levels. By referring to Figure 2, we can see that there are conflicting requirements for the readout circuit and the sensor.

It is well known that the main cause of MOSFET mismatch in the subthreshold region is variations in the threshold voltage. The threshold voltage of a MOSFET is determined by the oxide thickness and doping concentration of the substrate region (see Equations (3) and (4)). The mismatch in Vt is caused by the variation in the number of charged ions in the depletion layer. These fluctuations are reduced by increasing the doping level. The standard deviation of a single MOSFET threshold voltage, with an area of W*L, can be calculated using the Pelgrom parameter [20]:

(5) $$\sigma_{\Delta V_{t}}=\frac{\sqrt[{4}]{4\phi_{F}q^{3}N_{a}\varepsilon_{0}\varepsilon_{ox}}}{C_{ox}\sqrt{WL}}=\frac{A_{V_{T}}}{\sqrt{2WL}}\;$$

where $A_{V_{T}}$ is the process mismatch parameter and *W* and *L* are the transistor’s channel width and length, respectfully.

By decreasing the oxide thickness and increasing the doping level, the mismatch is reduced. For a typical process of 130 nm CMOS–SOI $A_{V_{T}}\;$= 5.4 mV*μm for a standard NMOS device. Assuming large area transistors of $\frac{W}{L}=18\cdot \frac{4\cdot 9.9\;\mu m}{5\cdot 9.5\;\mu m}$ corresponding to this study the standard deviation in room temperature is 0.02 mV.

## 6. Conclusions

GMOS sensors are silicon MEMS devices that are cost efficient, have low power consumption and a small form factor. By adding an analog readout connected to an ADC (analog to digital converter), we can achieve the digital outcomes, such as digital signal processing, wireless communication and making our smart sensor smarter by adding ML/AI (machine learning/artificial intelligence).

Since there are conflicting technological requirements for the sensor and the readout circuitry, we recommend retracting the monolithic system-on-a-chip approach. This approach has been the ambitious goal of the sensor community for the last 20 years. The chiplet approach is now recommended as the best approach.

MEMS sensors are currently undergoing a fourth paradigm shift:

- The first-generation scientific and industrial MEMS sensors, such as inertial sensors, were stand-alone units;
- The second-generation MEMS sensors were integrated with their readout circuitry to yield digital MEMS sensors;
- The third generation included smart sensors connected to the cloud for post-processing using AI and machine learning techniques;
- The latest generation is intelligent sensors that utilize advanced processing units. The intelligent sensing modules of these sensors rely on data measured by the sensor. Since the data will always include noise and false measurements, it is important to be mindful of this and to use various statistical approaches and estimators, which has become part of the research.

We recommend applying the best technology for each challenge, and not necessarily integrating everything.

## Figures and Tables

**Table 1:** Sensitivity (actual and normalized to gain) at heater voltage of 2.5 V (190 °C).

Simulated Gain	2	11	22	132
sensitivity, mV/ppm	0.243	1.520	2.730	16.795
actual gain	1.92	11.68	21.58	131.25
Sense/gain, mV/ppm	0.127	0.130	0.127	0.128

**Table 2:** Sensitivity (actual and normalized to gain) at heater voltage of 3 V (240 °C).

Simulated Gain	2	11	22
sensitivity, mV/ppm	0.619	3.64	5.95
actual gain	1.92	11.68	21.58
Sense/gain, mV/ppm	0.322	0.317	0.276

**Table 3:** Comparison of GMOS and practical industry gas sensors.

Gas Sensors Comparison	Sensor Type	Target Gas	Range	Resolution	Power Consumption
Figaro TGS 2620	MOS	Alcohol, Solvent apors	50–5000 ppm ethanol	5–10%	<225 mW
Winsen MQ-138	Semiconductor	Toluene, Acetone, Alcohol, Hydrogen	5–500 ppm	5–10%	<950 mW
Sensirion SPG40	*Mox	*VOC	0–1000 ppm ethanol equiv.	<10%	<12 mW
GMOS (this work)	MOSFET-MEMS	*VOC	0–10,000 ppm ethanol	1–5%	<10 mW

*MOx—metal-oxide; *VOC—volatile organic compound. Note: Resolution was calculated in percentage according to the sensors’ datasheets.

## Data Availability

The original contributions presented in this study are included in the article. Further inquiries can be directed to the corresponding author.
